# Biogeographical diet variation within and between the rabbitfishes *Siganus corallinus*, *Siganus doliatus*, *Siganus trispilos* and *Siganus virgatus*

**DOI:** 10.1002/ece3.11326

**Published:** 2024-06-17

**Authors:** Salvador Zarco‐Perello, Storm B. Martin, Andrew Hoey

**Affiliations:** ^1^ Harry Butler Institute, Murdoch University Perth Western Australia Australia; ^2^ College of Life Sciences and Agriculture University of New Hampshire Durham New Hampshire USA; ^3^ College of Science and Engineering James Cook University Townsville Queensland Australia

**Keywords:** coral reefs, herbivory, inter‐specific variation, intra‐specific variation, trophic niche, trophic plasticity

## Abstract

Feeding habits of herbivorous fishes play an important role in shaping the form and function of coastal marine ecosystems. Rabbitfishes (Siganidae) are important consumers of macroalgae on Indo‐West Pacific coral reefs. However, it is unclear how their diet varies among and within species at biogeographical scales, casting doubt on their precise functional roles across different regions. The present study assessed the inter‐ and intra‐specific diet variation of four rabbitfishes (*Siganus trispilos, Siganus corallinus, Siganus virgatus* and *Siganus doliatus*) factored by morphological relatedness among populations from Ningaloo Reef (western Australia), the Great Barrier Reef (GBR, eastern Australia) and the Yaeyama Islands (Okinawa Prefecture, Japan). Results showed that the region had a strong effect on diet, effectively reducing the expected effect of morphologic similitude. While intra‐specific differences were only significant when populations inhabited different regions; interspecific differences were not as predicted, with different morphotypes having similar diets when populations inhabited the same regions. Rabbitfishes consumed more corticated and filamentous macroalgae on the GBR, more foliose and membranous macroalgae at the Yaeyama Islands, and more leathery macroalgae at Ningaloo Reef. The findings indicate that rabbitfishes have high diet plasticity, and hence their functional role as mediators of competition between macroalgae and corals can change across biogeographic regions. Local context is therefore important when assessing the diet and functional role of herbivorous fishes. As climate change unfolds, shifts in the distribution, trophic behaviour and function of species are expected, making the study of trophic plasticity more important.

## Graphical abstract

The present study assessed the inter‐and intra‐specific diet variation of rabbitfishes factored by morphological relatedness among populations from western Australia, the Great Barrier Reef and Okinawa. Results showed that region had a strong effect on diet, effectively reducing the expected effect of morphologic similitude, indicating that rabbitfishes possess high diet plasticity, and hence their functional role as mediators of competition between macroalgae and corals can change across biogeographic regions. Local context is therefore important for an accurate assessment of the diet and functional role of herbivorous fishes.

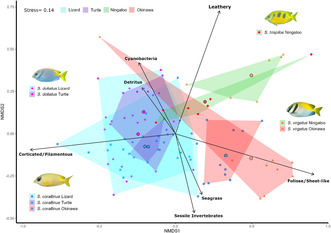

## 1 INTRODUCTION

Primary consumption is an important ecological process that greatly influences energy flow and habitat structure in natural ecosystems. Herbivores link primary producers and higher‐order consumers, initiating the transfer of energy across the trophic network and sustaining, directly and indirectly, the secondary productivity of higher trophic levels (Zarco‐Perello et al., 2019). Herbivores can consume the majority of primary productivity, and influence the standing biomass and composition of benthic communities (Spadaro & Butler, 2021). On coral reefs, fish species of the families Acanthuridae (surgeonfishes) and Siganidae (rabbitfishes), as well as the subfamily Scarinae (parrotfishes), are conspicuous and abundant consumers of macroalgae, and have been suggested to act as ecosystem engineers (Steneck et al., 2017). It is generally theorised that abundant and functionally diverse communities of herbivorous fishes strengthen the resilience of coral‐dominated states by preventing phase‐shifts to macroalgal‐dominated states following disturbances such as cyclones or high temperatures that cause mass coral bleaching (Cheal et al., 2013); however, this will be dependent on the identity and relative abundances of the macroalgae and herbivorous fish species present in each reef system (Bellwood et al., 2006; Puk et al., 2020). Inter‐species comparisons of diet and feeding behaviour are widespread among all the most important families of herbivorous fishes, delineating functional diversity and redundancy within fish communities (Johansson et al., 2013; Kelly et al., 2016). Species that feed substantially on leathery macroalgae (browsers) can shorten the algal canopy, enhancing light penetration to the benthos and reducing physical damage to coral colonies (McCook et al., 2001), whereas consumers of short foliose and filamentous macroalgae (grazers) can clear benthic space and potentially facilitate coral recruitment (Heenan et al., 2016; Korzen et al., 2011). Generally, although there are often many species of herbivorous fishes that are grazers, typically only a few species have been identified as important browsers in reef systems (Puk et al., 2016).

The specific diet of different herbivorous fishes has been linked to morpho‐functional adaptations. Parrotfishes are clearly distinguished from other families by their beak‐like fused teeth, which allows them to not only consume turf and cyanobacteria (Nicholson & Clements, 2023) but also excavate deep into the substratum and scoop sediment, detritus and calcium carbonate; explaining the lack of species that feed on fleshy macroalgae, with a few exceptions in the genera *Sparisoma, Calotomus* and *Leptoscarus* (Bonaldo et al., 2014). Surgeonfishes exhibit a greater diversity of morpho‐functional adaptations for feeding specialisations. For instance, browsers of large leathery macroalgae are restricted to a few species of the genus *Naso* with teeth adapted to perforate and rip the algal thallus, whereas consumers of detritus and filamentous macroalgae are grouped in the genus *Ctenochaetus*, who evolved brush‐like teeth which allows them to collect particulate material from turf mats (Tebbett et al., 2022). Rabbitfishes are a particular case, because all species are grouped within the genus *Siganus* spp; nevertheless, morphological and behavioural adaptations differentiate species in their trophic ecology, and rabbitfishes collectively exhibit substantial functional significance and variation in diet comparable to bigger families of herbivorous fish (Bellwood, Hoey, et al., 2014; Hoey et al., 2013). For instance, flat‐snouted species such as *Siganus canaliculatus* and *S*. *virgatus* have been identified as important browsers in Orpheus Island on the Great Barrier Reef (GBR; Bennett & Bellwood, 2011; Fox & Bellwood, 2008) and coral reefs of the Indo‐Pacific region, respectively (Bauman et al., 2017; Müller et al., 2021; Plass‐Johnson et al., 2015; Seah et al., 2021). On the other hand, species with more slender bodies and elongated snouts, such as *S. corallinus* and *S. vulpinus*, have been identified as important consumers of filamentous algae and cyanobacteria in open and cryptic reef spaces, allowing them to access unique trophic resources in the ecosystem (Brandl & Bellwood, 2014; Fox & Bellwood, 2013).

Macroalgae consumption by herbivores may not only depend on fish species identity but may also vary across habitats and biogeographic distances. Fishes are regularly classified into trophic guilds, but feeding behaviours and prey items can change depending on the environmental and biological factors in different locations. Among fish, species of different trophic levels have shown feeding plasticity across space (Hamilton et al., 2011), even in species typically considered to have specialised diets. Such as the corallivorous butterflyfish *Chaetodon octofasciatus*, whose populations can differ significantly in the number of coral species eaten and the number of bites taken by each of them (Feary et al., 2018). Herbivorous species are most likely not the exception; however, diet plasticity has rarely been assessed for herbivorous coral reef fishes, particularly at biogeographical scales. Among the few studies that have examined spatial variation in feeding behaviour, Wilson et al. (2021) found significant variation in bite rates for the parrotfishes *Scarus vetula* and *Sparisoma viride* between coral reefs in Barbuda, Antigua and Bonaire, and Locham et al. (2015) found the parrotfish *Leptoscarus vaigiensis* differed in diet diversity and breadth across coral reefs in Kenya. Similarly, Duran et al. (2019) found that the surgeonfishes *Acanthurus coeruleus* and *A. tractus* took different proportions of bites on different food items, such as turf, epiphytes, or sessile invertebrates among coral reefs of the Florida Keys. The spatial variability in the trophic ecology of rabbitfishes has only been assessed within the Great Barrier Reef, where diet analyses indicated clear inter‐specific differences; however, a formal assessment of intra‐specific variability has not yet been done (Hoey et al., 2013). Thus, it is unclear how their consumption of algae and their functional role, might vary among and within species at large biogeographical scales. Addressing this knowledge gap is important because coral reef ecosystems are managed at the local level, and it is necessary to understand the spatial variability of the ecological processes related to their stability. If herbivorous fishes change their feeding activity dramatically among regions, their functional role will likewise change, impacting the capacity of the reef to suppress or recover from changes in the benthic community.

**FIGURE 1:**
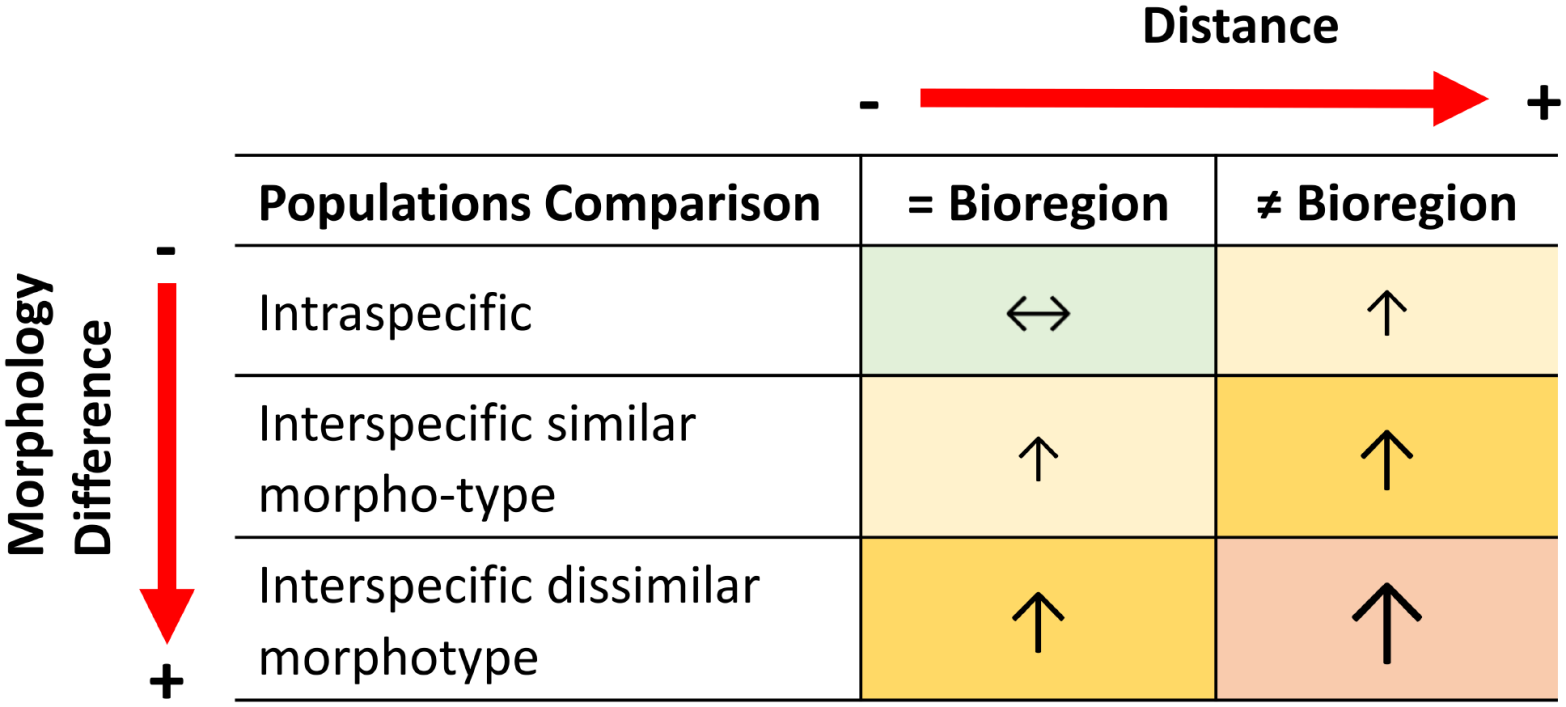
Hypothesised magnitudes of dissimilitude (size of arrows and colours) in diet within (intra‐specific) and among (interspecific) species of rabbitfish as a factor of shared geographic distribution and morphologic similitude.

The present study aimed to shed light on the inter‐ and intra‐specific diet variation factored by morphological similitude and biogeographic region by comparing populations of closely related species of rabbitfishes (*Siganus trispilos, Siganus corallinus, Siganus virgatus* and *Siganus doliatus*) within and among regions separated by thousands of kilometres: Ningaloo (western Australia), the Great Barrier Reef (GBR, eastern Australia) and Okinawa (Japan). We hypothesised that: (1) morphologically similar species (long/short snout) should have more similar diets and functional roles; however, (2) intra‐ and inter‐specific differences will increase between populations of different biogeographic regions due to different physical and biological environments (Figure 1).

## 2 METHODS

### 2.1 Locations and species of study

**FIGURE 2:**
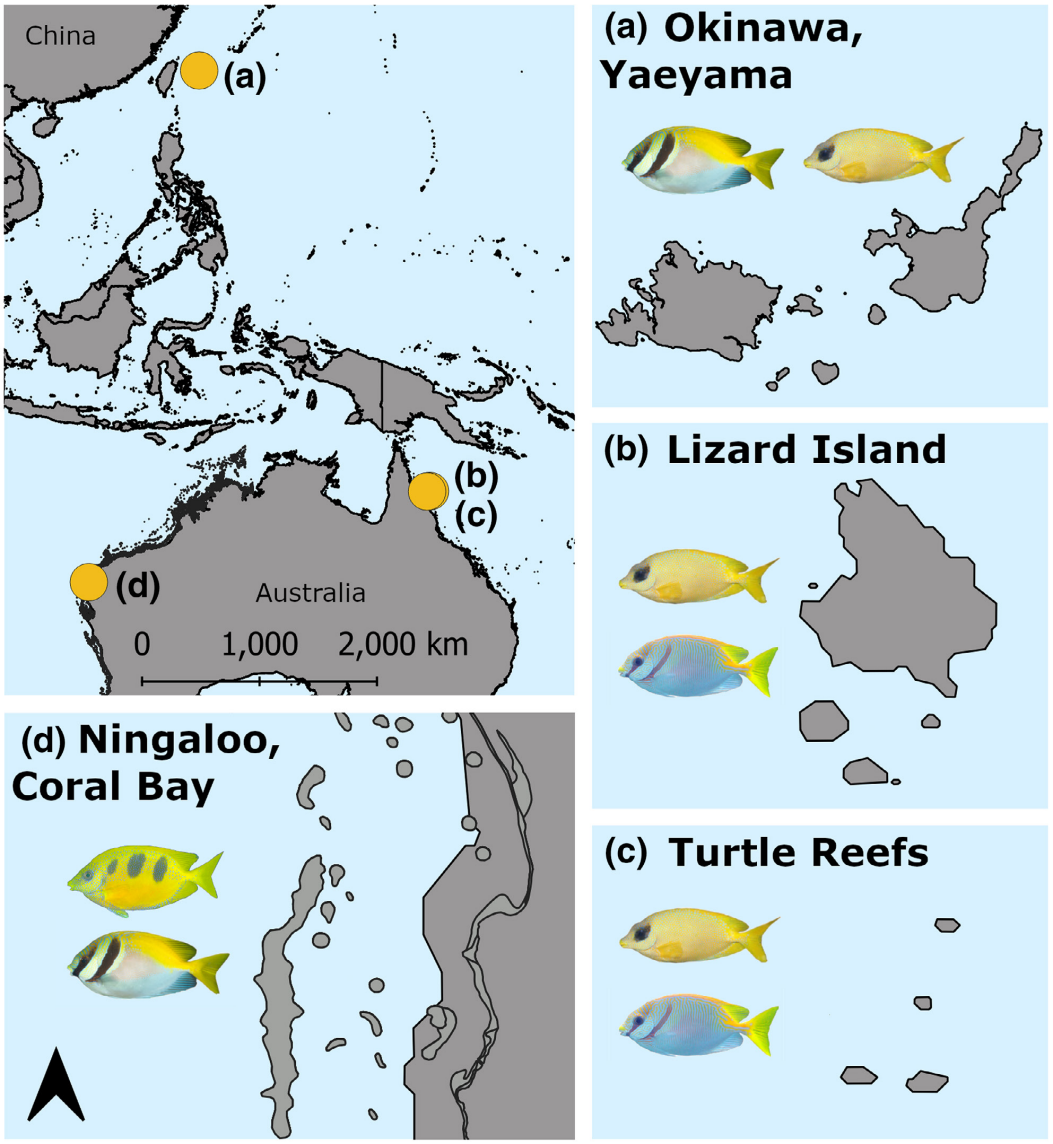
Sampling locations of rabbitfishes for diet comparisons. (a) Yaeyama Islands, Okinawa (*S*. *corallinus, S*. *virgatus*); (b) Lizard Island, mid‐shelf northern Great Barrier Reef (*S*. *corallinus, S*. *doliatus*); (c) Turtle Group, inner‐shelf northern Great Barrier Reef (*S*. *corallinus, S*. *doliatus*); and (d) Ningaloo Reef (*S*. *trispilos, S*. *virgatus*).

The study focused on four closely related species of rabbitfish (*Siganus virgatus, S. doliatus, S. corallinus* and *S. trispilos*; Siqueira et al., 2019) that have populations in three regions set apart by thousands of kilometres: Okinawa (Yaeyama Islands), the Great Barrier Reef (GBR; Turtle Group and Lizard Island) and Ningaloo Reef (Coral Bay; Figure 2). *Siganus virgatus* and *S*. *doliatus* have short snouts and are considered sister species based on phylogenetic analyses (Siqueira et al., 2019). *Siganus corallinus* and *S*. *trispilos* have longer snouts and based on morphological similarities, are thought to be sister species (Woodland & Allen, 1977), although their relatedness has yet to be confirmed by genetic analyses. *Siganus virgatus* and *S*. *doliatus* are distributed across the Indo‐West Pacific and Western Pacific, respectively, and have been observed regularly biting from assays of *Sargassum* spp. (e.g., Fox & Bellwood, 2013), although whether they are targeting the *Sargassum* itself or epibiota is unknown. *Siganus corallinus* is distributed across the Indo‐West Pacific and has been described as an ‘algal cropper’ (Hoey et al., 2013); *Siganus trispilos* is endemic to northwestern Australia and its diet has not yet been described, but we hypothesise that it should be functionally similar to the morphologically similar *S. corallinus* (Fox & Bellwood, 2013).

### 2.2 Diet information

Specimens of *S*. *virgatus* and *S. trispilos* were collected by spear [Murdoch University ethics permit number R3349/21 and Department of Fisheries (DPIRD) exemption 3699] between 9 am and 11 am in the backreef and lagoon habitats of Bateman (23°02′28″S, 113°47′04″E), Five Fingers (23°10′54″S, 113°45′51″E) and Yalobia (23°12′22″S, 113°45′33″E) reefs, Ningaloo Reef, Western Australia, during June 2022 and March–April 2023 (Table 1). Captured specimens were euthanised, stored in ice and dissected fresh at the Coral Bay Research Station (Murdoch University). The alimentary tract was removed, dissected open and the gut content preserved in a solution of 80% ethanol. Diet data from these samples were collected in the laboratory using a stereo microscope, similarly to previous studies (Nanami, 2018). Food items were spread evenly over a petri dish divided into a grid of 100 cells, identified to the lowest taxonomic level possible and assigned a proportion of the total gut content based on the proportional number of grid squares covered using the program ImageJ (rsb.info.nih.gov/ij/). Macroalgae in the diet were grouped into five morpho‐functional groups: foliose, membranous, corticated, filamentous and leathery. Categories other than macroalgae comprised seagrass, cyanobacteria, sessile invertebrates and detritus. Diet information for the populations of rabbitfishes from the GBR, was obtained from a diet database created by Hoey et al. (2013). Diet data from Okinawa were obtained from Nanami (2018) using the software Datathief III (Flower et al., 2016; Table 1).

**TABLE 1:** Number of specimens and body size of rabbitfishes included in the present study.

Species	Region	*N*	Fork length (mm)	References
*S. corallinus*	Okinawa, Yaeyama Islands	10	126–205	Nanami (2018)
*S. virgatus*		10	159–215	
*S. corallinus*	GBR mid‐shelf (Lizard Island)	22	183–234	Hoey et al. (2013)
	GBR inner‐shelf (Turtle Group)	6		
*S. doliatus*	GBR mid‐shelf (Lizard Island)	20	165–250	
	GBR inner‐shelf (Turtle Group)	11		
*S. trispilos*	Ningaloo, Coral Bay	7	217–269	This study
*S. virgatus*		6	220–257	

Abbreviations: GBR, Great Barrier Reef; *N*, number of specimens collected.

### 2.3 Statistical analysis

Differences in diet between species and populations across regions were analysed with non‐metric multidimensional scaling (NMDS) based on Bray‐Curtis distances using the functions *vegdist* and *metaMDS* of the R package vegan (Oksanen et al., 2022). Statistical differences were tested with permutational analysis of variance (PERMANOVA), considering ‘region’ and ‘taxa’ with sister species nested in 2 levels (‘*S*. *virgatus* + *S*. *doliatus’* and ‘*S*. *trispilos* + *S*. *corallinus’*) as fixed factors, using the function *adonis2* of the R package vegan (Oksanen et al., 2022), followed by pairwise comparisons among species‐region combinations with adjusted p‐values based on the Hommel method using the functions *pairwise.adonis2* and *p.adjust.m* of the R package pairwiseAdonis (Martinez, 2017).

## 3 RESULTS

The diets of rabbitfishes differed among species and geographic location, with most of the variance explained by region (PERMANOVA; region: pseudo‐*F*_3,83_ = 27.0833, *p* = .0001; taxa: pseudo‐*F*_1,83_ = 4.9176, *p* = .0001; species: pseudo‐*F*_2,83_ = 3.0200, *p* = .0138; Table S1). Every pairwise comparison of rabbitfish populations between regions, both intra‐specific and inter‐specific, yielded statistically significant differences. However, within regions, no significant differences were detected, except for interspecific comparisons at Turtle Group and Lizard Island in the GBR (Table S2).

### 3.1 Interspecific differences in diet

**FIGURE 3:**
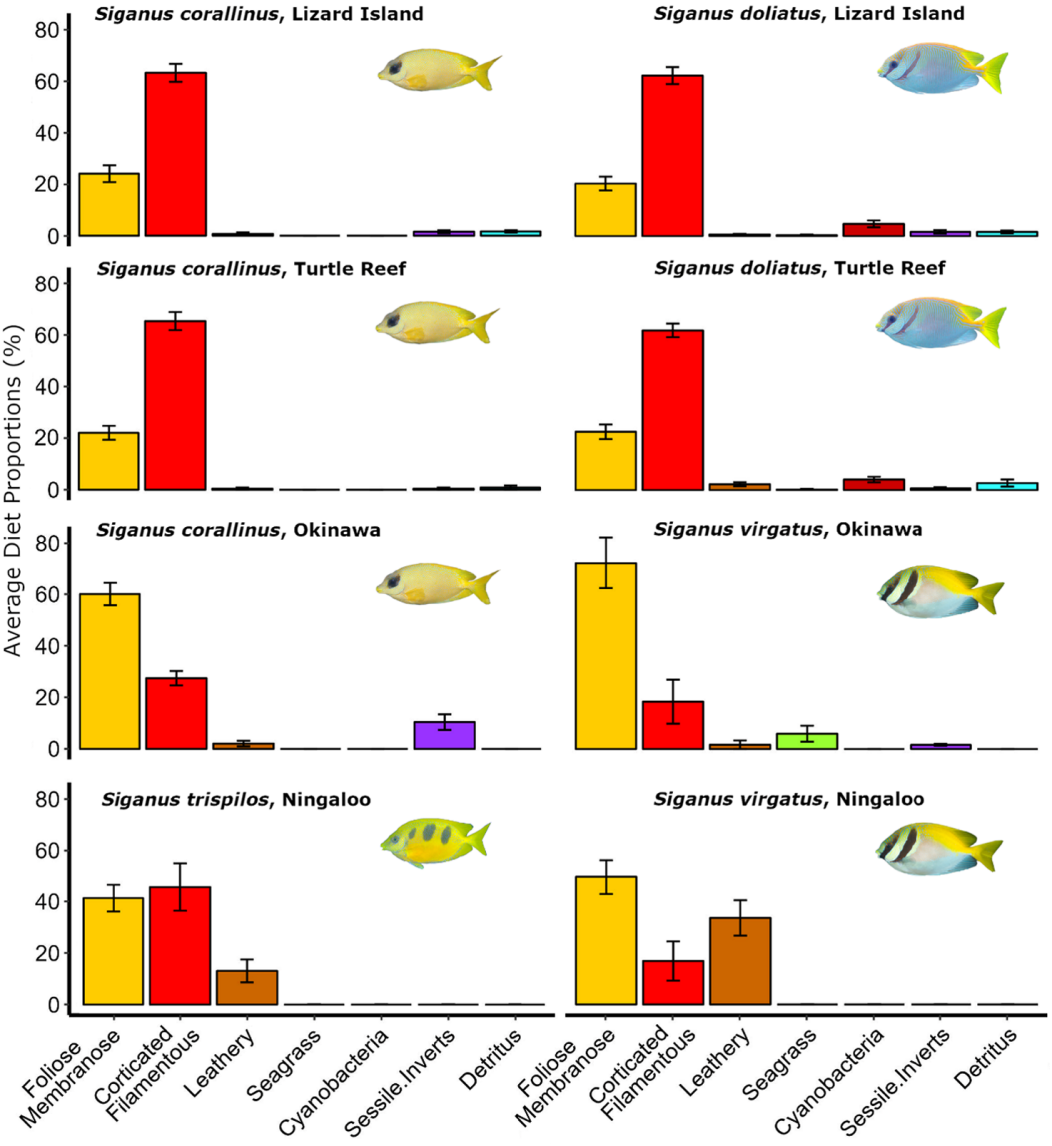
Variation in the gut contents of populations of the rabbitfishes *Siganus corallinus, S. doliatus, S. virgatus and S. trispilos* from different regions of the world: Great Barrier Reef (Turtle Group and Lizard Island), Western Australia (Ningaloo) and Japan (Okinawa). Barplots show means and standard errors (SE) of different diet items.

The diet of *S*. *corallinus* from Lizard Island was significantly different from all other species of rabbitfishes. Whereas *S. corallinus* from Turtle Group (inner‐shelf GBR) differed in diet from all populations but *S. doliatus* from Lizard Island (mid‐shelf GBR). Similarly, the diet of *S. corallinus* from Okinawan reefs differed from all populations apart from *S. virgatus* inhabiting the same region. Overall, *S. corallinus* and *S. doliatus* from the GBR had very similar diets dominated by corticated and filamentous algae (63.2 ± 3.4%, mean ± se), followed by foliose and membranous macroalgae (24.0 ± 3.2%). Okinawan rabbitfishes also had similar diets but with an inverse pattern, having higher proportions of foliose and membranous macroalgae (~60.1 ± 4.3%), and less corticated and filamentous algae (~27.4 ± 2.8%; Figure 3). *S. trispilos* from Ningaloo had a diet with equal proportions of foliose/membranous (41.3 ± 5.1%) and corticated/filamentous algae (45.6 ± 9.1%), followed by leathery macroalgae (13.1 ± 4.4%; Figure 3). This dietary composition made it significantly different from all the other rabbitfishes of other regions, except for *S. virgatus*, also from Ningaloo, which had a high proportion of leathery macroalgae (33.6 ± 6.8%), followed by foliose/membranous (49.5 ± 6.7%) and corticated/filamentous algae (16.9 ± 7.6%; Figure 3).

### 3.2 Intra‐specific differences in diet

**FIGURE 4:**
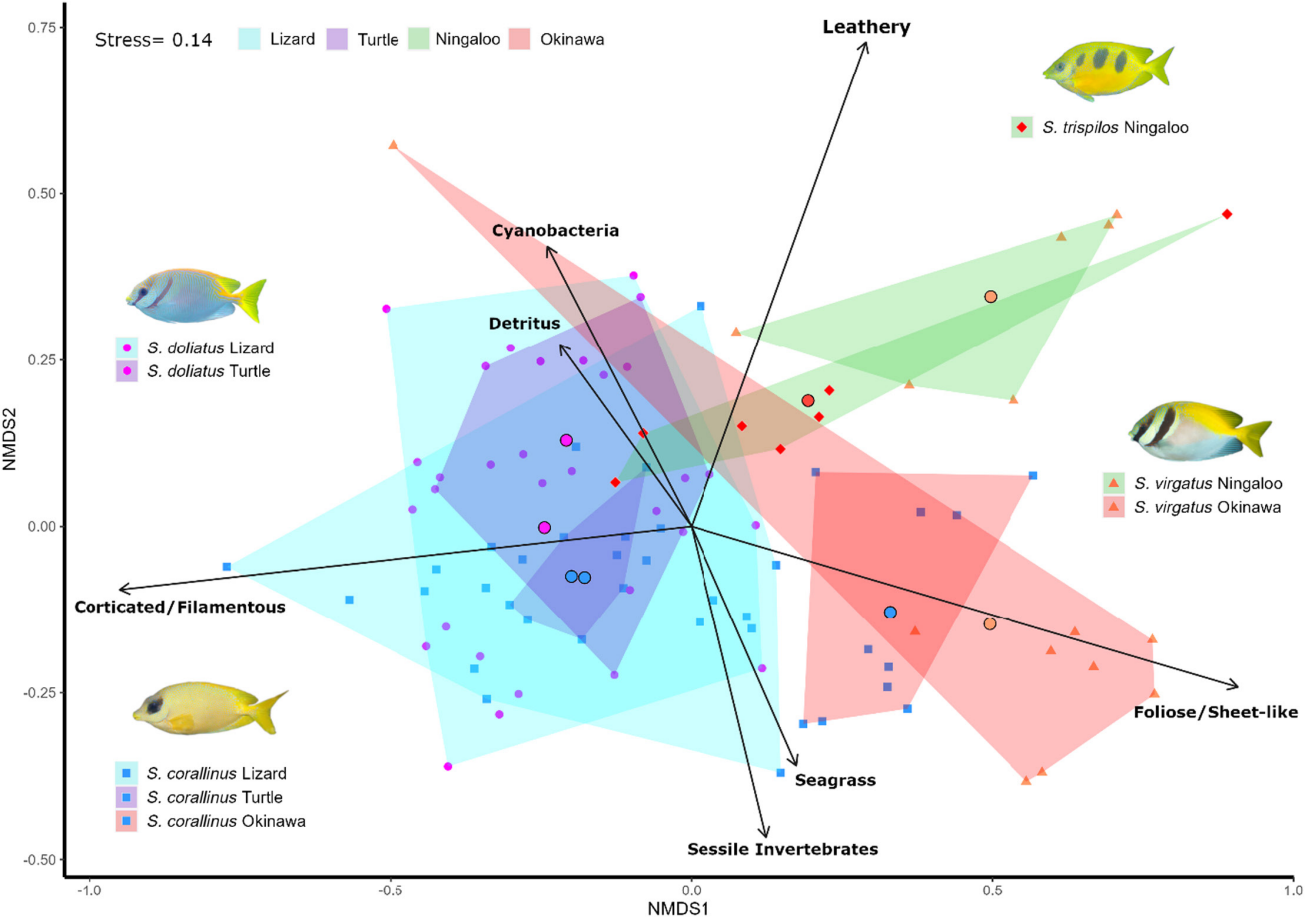
Non‐metric multidimensional scaling ordination showing differences in diet composition between the rabbitfishes *Siganus corallinus, S. doliatus*, *S. virgatus* and *S. trispilos* from different regions of the world: Great Barrier Reef (Turtle Group and Lizard Island), Western Australia (Ningaloo) and Japan (Okinawa). Bigger dots represent centroids of each population.

The diets of conspecific populations within the same region were not significantly different (Tables S1 and S2). Populations of *S. corallinus* from Lizard Island and Turtle Group within the GBR did not differ significantly in their diets. Likewise, populations of *S. doliatus* within these two locations in the GBR had similar diets. These populations had diets dominated by corticated and filamentous algae, seconded by foliose and membranous macroalgae; however, populations from the Turtle Group generally displayed less variation in diet among individuals than those from Lizard Island (Figure 4). In contrast, intra‐specific differences in the diet of rabbitfishes separated at continental scales were highly significant (Tables S1 and S2). Both populations of *S. corallinus* from the GBR had significantly different diets than those from Okinawa, which tended to feed more on foliose and membranous macroalgae and sessile invertebrates than individuals from the GBR (Figures 3 and 4). Likewise, populations of *S. virgatus* from Ningaloo and Okinawa also had marked differences in their diet, driven by higher consumption of leathery macroalgae by Ningaloo individuals and higher diet proportions of foliose and membranous macroalgae and seagrass by individuals from Okinawa (Figures 3 and 4).

## 4 DISCUSSION

We hypothesised that morphologically similar species would have similar diets, and that intra‐ and inter‐specific differences would be greater between populations of different biogeographic regions. We found a strong effect of region on diet, explaining 46% of the variation, effectively reducing the expected effect of morphologic resemblance. While intra‐specific differences were only significant when populations inhabited different regions as expected, interspecific differences were not as predicted, with different species having similar diets when populations inhabited the same regions.

The significant biogeographic variation in diet of these rabbitfishes can be attributed to high trophic plasticity, enabling an adaptive response to local resource availability. Resource availability is among the most important factors determining foraging plasticity in fish populations. The diet of fish species can vary depending on the interaction between the relative abundance and relative preference of dietary resources (Barrientos et al., 2021). Such examples have been observed in some surgeonfish species, which usually prefer filamentous turf algae but consume more fleshy and calcareous macroalgae at locations with greater abundance of these resources (Francini‐Filho et al., 2010). The influence of resource availability on the diet of rabbitfishes has only been documented in range‐extending species: *S*. *luridus* and *S*. *rivulatus* in the Mediterranean Sea and *S*. *fuscescens* in western Australia, which consume a great variety of macrophytes in temperate reefs that are not found in their original habitats in coral reefs (Azzurro et al., 2007; Bariche, 2006; Zarco‐Perello et al., 2019).

Our results document the trophic plasticity of rabbitfishes within tropical regions at biogeographical scales and may reflect the availability of dietary resources in each location. At Ningaloo Reef, algal communities are dominated by turfs; however, extensive beds of the canopy‐forming macroalgae *Sargassum* spp. occur within backreef habitats (Evans et al., 2014; Wilson et al., 2014). On the Great Barrier Reef, there is considerable cross‐shelf variation in benthic communities, with macroalgae rare or absent on mid‐shelf reefs (especially canopy‐forming macroalgae), yet accounting for upwards of 50% of the benthic community on inner‐shelf reefs (Hoey & Bellwood, 2010; Wismer et al., 2009). Indeed, the cover of fleshy macroalgae was generally low on the mid‐shelf reefs surrounding Lizard Island at the time of sampling, whereas macroalgal cover was generally greater and more variable on the inner‐shelf reefs of the Turtle Group (Hoey & Bellwood, 2010; Wismer et al., 2009). Likewise, there is considerable variation in benthic composition among coral reefs on the Yaeyama Islands, with most dominated by scleractinian corals and bare rock, and some (2 of 63 sampling sites) with a high cover of macroalgae (e.g., *Padina* spp. and *Sargassum* spp.; Nanami, 2018). Reefs in Nagura Bay and Sekisei Lagoon used to be composed of massive and encrusting corals, and branching Acropora communities; however, these have suffered high mortalities following bleaching events in 1998, 2001, 2003 and 2007, most likely leading to a high cover of turf and small foliose macroalgae on the reef surfaces, like the diet of rabbitfishes at this location (Roeroe et al., 2013). The beds of *Sargassum* spp. in back reef habitats at Ningaloo Reef may explain why the gut content of *S*. *virgatus* and *S. trispilos* from this region had a high proportion of leathery macroalgae; however, it does not explain the lack of leathery macroalgae in the gut content of *S. corallinus* and *S. doliatus* from inner‐shelf reefs of the GBR (i.e., Turtle Group). The diet of rabbitfishes likely reflects the relative availability and palatability of potential dietary resources within their home ranges, and highlights the dangers of inferring diet from algal assays, even when those algae are naturally abundant (Bauman et al., 2017; Müller et al., 2021; Plass‐Johnson et al., 2015; Seah et al., 2021).

The availability and palatability of dietary resources can interact with other habitat factors, such as topographic complexity and the risk of predation, to influence an individual's diet. Populations living in habitats with different structural complexity can differ in rates of consumption of different macroalgal resources (Vergés et al., 2011), including for the rabbitfishes *S*. *doliatus* and *S*. *canaliculatus* on the GBR, whose populations differed in macroalgae consumption among the reef slope and reef crest, the latter having higher topographic complexity (Loffler et al., 2015). Habitat complexity can also interact with predator‐prey dynamics and have important impacts on the foraging behaviour of herbivores, where predation risk can suppress herbivory more effectively in topographically complex areas; thus, populations residing in ecosystems with differences in predator densities and composition can suffer significant changes in macroalgae consumption (Catano et al., 2016). For instance, the foraging of *S. virgatus* and *S. javus* in the coral reefs of Singapore was heavily affected by the presence of a predator decoy, significantly reducing their consumption of *Sargassum* spp. (Bauman et al., 2019).

Consumption of new resources could be limited by the morphological and physiological traits of each species (Bellwood, Hoey, et al., 2014). We expected the flat‐snouted, robust species (*S*. *doliatus* and *S*. *virgatus*) would consistently consume important amounts of fleshy and leathery macroalgae, and that species with elongated snouts and slender bodies (*S*. *corallinus* and *S*. *trispilos*) would feed mostly on filamentous algae, consistent with previous literature (Brandl & Bellwood, 2014; Fox & Bellwood, 2013). However, all species ingested a mix of the same macroalgae types, similar to previous findings for some surgeonfishes, where no relationship was found between morphology and diet specialisation (Brandl et al., 2015). The substantial amounts of leathery macroalgae in the gut content of *S*. *trispilos* show that a longer snout does not exclude consumption of tough macrophytes or restrict the trophic behaviour of rabbitfishes with this morphology to grazing. Rather, a long snout extends their trophic niche inside and outside cryptic reef spaces (Brandl & Bellwood, 2014; Fox & Bellwood, 2013). Dentition traits are important for the abilities of animals to consume certain types of food (Bellwood, Goatley, et al., 2014), and although no study has conducted a detailed analysis of dentition among rabbitfish species, they seem to have similar traits, comprised of narrow incisor‐like bicuspid or tricuspid teeth (Woodland, 1990). Our results indicate that they may be suitable to crop a wide variety of resources.

The trophic plasticity of rabbitfishes has important repercussions for their functional roles in marine ecosystems. Herbivory can reinforce the stability and resilience of coral dominated states in tropical reefs, but its effectiveness is determined by the suite of specific trophic behaviours of the herbivorous community (Hoey & Bellwood, 2009). Currently, it is common practice to assign herbivorous fishes to trophic guilds based on studies conducted in just a few locations (Edwards et al., 2014). Our results show the importance of local herbivory assessments and indicate that the behavioural plasticity of species must be considered when assessing the intensity of different herbivory functions at broad biogeographic scales. In similitude to the concepts of the fundamental versus realised niche, our study highlights the distinction between (a) *fundamental herbivory*: the ability of a species to perform herbivory functions and (b) *realised herbivory*: the function executed by one population under specific physical and biological conditions. For instance, the batfish *Platax pinnatus* normally feeds on benthic and planktonic invertebrates, but consumed high amounts of *Sargassum* sp. when it was extraordinarily presented with this resource in a herbivory‐exclusion experiment (Bellwood et al., 2006). Moreover, it is important that local studies collect enough samples that capture the full extent of the trophic niche. In our study, diet estimations for *S*. *trispilos* and *S*. *virgatus* from Ningaloo Reef and *S. corallinus* from the Turtle Group reefs were limited (*n* = 6–7 individuals), and as such they should be interpreted with some caution. Nevertheless, the gut content for these sample populations was largely consistent among individuals and is therefore likely reflective of their trophic niche. For instance, *S. corallinus* from the Turtle Group reefs had diet proportions and a niche centroid very close to the other rabbitfish populations from the Great Barrier Reef, but the extent of their trophic niches was disparate because the latter had bigger sample sizes. Thus, the trophic functions of the fish community must be carefully assessed at local scales to ensure that the species responsible for critical ecosystem processes are accurately identified and included in management strategies (Chung et al., 2019).

Our study explored the differences in diet between populations of rabbitfishes across biogeographic distribution and morphology. We found significant trophic plasticity among the four rabbitfish species compared, and diets appeared to be strongly related to region. The results suggest that the trophic role among rabbitfishes, and potentially species of other herbivorous fish families, is difficult to extrapolate across locations and across species, even when they are closely related. These results are particularly important as the assessment of coral reef resilience includes examining the abundance of key fish herbivorous guilds that can prevent and revert phase shifts based on which macroalgae they consume. As climate change unfolds, shifts in the distribution, trophic behaviour and function of species are expected, making the study of trophic plasticity more important.

## AUTHOR CONTRIBUTIONS

**Salvador Zarco‐Perello:** Conceptualization (lead); data curation (lead); formal analysis (lead); funding acquisition (equal); investigation (lead); writing – original draft (lead). **Storm B. Martin:** Funding acquisition (equal); resources (equal); writing – review and editing (supporting). **Andrew Hoey:** Data curation (supporting); formal analysis (supporting); methodology (supporting); writing – review and editing (supporting).

## CONFLICT OF INTEREST STATEMENT

No conflicts of interest are declared by the authors.

## Supporting information


Data S1.

## Data Availability

Data available from the Dryad Digital Repository (Zarco‐Perello et al. 2024).
