# Supplementary material for: Biogeographical diet variation within and between the rabbitfishes Siganus corallinus, Siganus doliatus, Siganus trispilos and Siganus virgatus
Source: Ecol Evol. 2024 Jun 17;14(6):e11326. doi: 10.1002/ece3.11326 (PMC11183942; doi:10.1002/ece3.11326)
Supplement: Supplementary file 1 — Data S1. [file ECE3-14-e11326-s001.zip › Zarco_Perello Storm Hoey. Supplementary Materials_revised.docx]

Supplementary Materials

Biogeographical diet variation within and between the rabbitfishes *Siganus corallinus, Siganus virgatus, Siganus doliatus* and the endemic *Siganus trispilos*.

Zarco-Perello, S. ^1, 2^, Martin, S. ^1^, Hoey A.^3^

^1^ Harry Butler Institute, Murdoch University, Perth, Australia.

^2^ College of Life Sciences and Agriculture, University of New Hampshire, Durham, USA.

^3^ ARC Centre of Excellence for Coral Reef Studies and College of Science and Engineering, James Cook University, Townsville, Queensland, Australia

**Table S1. Results of the PERMANOVA, homogeneity of variances and pairwise comparisons in diet composition between different species and populations of the rabbitfishes Siganus trispilos, Siganus corallinus and Siganus virgatus.**

Model:

adonis2(formula = Sig.diet.dbs ~ Location + Sister.taxa / Species, data = Sig.names, permutations = 9999, strata = Sister.taxa)

General Anova:

Df SumOfSqs R2 F Pr(>F)

Location 3 2.6984 0.46374 27.0833 0.0001 ***

Sister.taxa 1 0.1633 0.02807 4.9176 0.0001 ***

Sister.taxa:Species 2 0.2006 0.03447 3.0200 0.0138 *

Residual 83 2.7565 0.47372

Total 89 5.8188 1.00000

Homogeneity of multivariate dispersions by populations

Df Sum Sq Mean Sq F N.Perm Pr(>F)

Groups 7 0.0785 0.0112140 1.3263 9999 0.242

Residuals 82 0.6933 0.0084549

Pairwise comparisons of populations:

$`S. virgatus Okinawa_vs_S. corallinus Okinawa`

Df SumOfSqs R2 F Pr(>F)

Population.ID 1 0.08738 0.10459 1.9858 0.138

Residual 17 0.74808 0.89541

Total 18 0.83547 1.00000

$`S. virgatus Okinawa_vs_S. trispilos Ningaloo`

Df SumOfSqs R2 F Pr(>F)

Population.ID 1 0.35606 0.31696 6.4967 0.009 **

Residual 14 0.76729 0.68304

Total 15 1.12335 1.00000

---

$`S. virgatus Okinawa_vs_S. virgatus Ningaloo`

Df SumOfSqs R2 F Pr(>F)

Population.ID 1 0.35164 0.33924 6.6744 0.007 **

Residual 13 0.68491 0.66076

Total 14 1.03655 1.00000

---

$`S. virgatus Okinawa_vs_S. corallinus Lizard`

Df SumOfSqs R2 F Pr(>F)

Population.ID 1 0.82694 0.42943 21.073 0.001 ***

Residual 28 1.09874 0.57057

Total 29 1.92568 1.00000

---

$`S. virgatus Okinawa_vs_S. corallinus Turtle`

Df SumOfSqs R2 F Pr(>F)

Population.ID 1 0.53068 0.43887 10.167 0.004 **

Residual 13 0.67852 0.56113

Total 14 1.20920 1.00000

---

$`S. virgatus Okinawa_vs_S. doliatus Lizard`

Df SumOfSqs R2 F Pr(>F)

Population.ID 1 0.93316 0.43822 21.062 0.001 ***

Residual 27 1.19626 0.56178

Total 28 2.12942 1.00000

---

$`S. virgatus Okinawa_vs_S. doliatus Turtle`

Df SumOfSqs R2 F Pr(>F)

Population.ID 1 0.79244 0.49278 17.488 0.001 ***

Residual 18 0.81565 0.50722

Total 19 1.60809 1.00000

---

$`S. corallinus Okinawa_vs_S. trispilos Ningaloo`

Df SumOfSqs R2 F Pr(>F)

Population.ID 1 0.22613 0.39694 9.8733 0.001 ***

Residual 15 0.34355 0.60306

Total 16 0.56968 1.00000

---

$`S. corallinus Okinawa_vs_S. virgatus Ningaloo`

Df SumOfSqs R2 F Pr(>F)

Population.ID 1 0.35591 0.57677 19.079 0.001 ***

Residual 14 0.26117 0.42323

Total 15 0.61708 1.00000

---

$`S. corallinus Okinawa_vs_S. corallinus Lizard`

Df SumOfSqs R2 F Pr(>F)

Population.ID 1 0.58283 0.46336 25.04 0.001 ***

Residual 29 0.67500 0.53664

Total 30 1.25783 1.00000

---

$`S. corallinus Okinawa_vs_S. corallinus Turtle`

Df SumOfSqs R2 F Pr(>F)

Population.ID 1 0.33051 0.61849 22.697 0.001 ***

Residual 14 0.20387 0.38151

Total 15 0.53438 1.00000

---

$`S. corallinus Lizard_vs_S. corallinus Turtle`

Df SumOfSqs R2 F Pr(>F)

Population.ID 1 0.01482 0.01912 0.4874 0.708

Residual 25 0.75990 0.98088

Total 26 0.77471 1.00000

---

$`S. corallinus Okinawa_vs_S. doliatus Lizard`

Df SumOfSqs R2 F Pr(>F)

Population.ID 1 0.67546 0.46648 24.482 0.001 ***

Residual 28 0.77252 0.53352

Total 29 1.44799 1.00000

---

$`S. corallinus Okinawa_vs_S. doliatus Turtle`

Df SumOfSqs R2 F Pr(>F)

Population.ID 1 0.55738 0.58715 27.021 0.001 ***

Residual 19 0.39192 0.41285

Total 20 0.94929 1.00000

---

$`S. trispilos Ningaloo_vs_S. virgatus Ningaloo`

Df SumOfSqs R2 F Pr(>F)

Population.ID 1 0.08403 0.2306 3.2968 0.077 .

Residual 11 0.28037 0.7694

Total 12 0.36441 1.0000

---

$`S. trispilos Ningaloo_vs_S. corallinus Lizard`

Df SumOfSqs R2 F Pr(>F)

Population.ID 1 0.39737 0.36403 14.883 0.001 ***

Residual 26 0.69421 0.63597

Total 27 1.09158 1.00000

---

$`S. trispilos Ningaloo_vs_S. corallinus Turtle`

Df SumOfSqs R2 F Pr(>F)

Population.ID 1 0.24380 0.47085 9.7879 0.006 **

Residual 11 0.27399 0.52915

Total 12 0.51779 1.00000

---

$`S. trispilos Ningaloo_vs_S. doliatus Lizard`

Df SumOfSqs R2 F Pr(>F)

Population.ID 1 0.43762 0.35598 13.819 0.001 ***

Residual 25 0.79173 0.64402

Total 26 1.22935 1.00000

---

$`S. trispilos Ningaloo_vs_S. doliatus Turtle`

Df SumOfSqs R2 F Pr(>F)

Population.ID 1 0.27230 0.37958 9.7892 0.003 **

Residual 16 0.44506 0.62042

Total 17 0.71736 1.00000

---

$`S. virgatus Ningaloo_vs_S. corallinus Lizard`

Df SumOfSqs R2 F Pr(>F)

Population.ID 1 0.90477 0.59658 36.97 0.001 ***

Residual 25 0.61183 0.40342

Total 26 1.51660 1.00000

---

$`S. virgatus Ningaloo_vs_S. corallinus Turtle`

Df SumOfSqs R2 F Pr(>F)

Population.ID 1 0.54308 0.79424 38.6 0.002 **

Residual 10 0.14069 0.20576

Total 11 0.68377 1.00000

---

$`S. virgatus Ningaloo_vs_S. doliatus Lizard`

Df SumOfSqs R2 F Pr(>F)

Population.ID 1 0.95829 0.57464 32.423 0.001 ***

Residual 24 0.70935 0.42536

Total 25 1.66763 1.00000

---

$`S. virgatus Ningaloo_vs_S. doliatus Turtle`

Df SumOfSqs R2 F Pr(>F)

Population.ID 1 0.65801 0.66685 30.024 0.001 ***

Residual 15 0.32874 0.33315

Total 16 0.98675 1.00000

---

$`S. corallinus Lizard_vs_S. doliatus Lizard`

Df SumOfSqs R2 F Pr(>F)

Population.ID 1 0.1202 0.08074 3.4257 0.017 *

Residual 39 1.3685 0.91926

Total 40 1.4887 1.00000

---

$`S. corallinus Lizard_vs_S. doliatus Turtle`

Df SumOfSqs R2 F Pr(>F)

Population.ID 1 0.15860 0.14556 5.1108 0.001 ***

Residual 30 0.93096 0.85444

Total 31 1.08956 1.00000

---

$`S. corallinus Turtle_vs_S. doliatus Lizard`

Df SumOfSqs R2 F Pr(>F)

Population.ID 1 0.06367 0.07426 1.9251 0.114

Residual 24 0.79381 0.92574

Total 25 0.85748 1.00000

$`S. corallinus Turtle_vs_S. doliatus Turtle`

Df SumOfSqs R2 F Pr(>F)

Population.ID 1 0.07112 0.1664 2.9943 0.03 *

Residual 15 0.35629 0.8336

Total 16 0.42742 1.0000

---

$`S. doliatus Lizard_vs_S. doliatus Turtle`

Df SumOfSqs R2 F Pr(>F)

Population.ID 1 0.02662 0.02685 0.8 0.557

Residual 29 0.96488 0.97315

Total 30 0.99150 1.00000

**Table S2. Synthesis of statistical significance (p-values from PERMANOVA) in pair-wise comparisons of differences in diet between species and populations of the rabbitfishes from the Great Barrier Reef (Turtle Reef and Lizard Island), Japan (Okinawa) and Western Australia (Ningaloo).**

| **Population** | *Siganus corallinus* Lizard | *Siganus corallinus* Turtle | *Siganus corallinus* Okinawa | *Siganus doliatus* Lizard | *Siganus doliatus* Turtle | *Siganus virgatus* Ningaloo | *Siganus virgatus* Okinawa |
| --- | --- | --- | --- | --- | --- | --- | --- |
| *S. corallinus* Turtle | **0.708** |  |  |  |  |  |  |
| *S. corallinus* Okinawa | ***  0.001 | ***  0.001 |  |  |  |  |  |
| *S. doliatus* Lizard | *  0.017 | **0.114** | ***  0.001 |  |  |  |  |
| *S. doliatus* Turtle | **  0.001 | *  0.03 | ***  0.001 | **0.557** |  |  |  |
| *S. virgatus* Ningaloo | ***  0.001 | **  0.002 | ***  0.001 | ***  0.001 | ***  0.001 |  |  |
| *S. virgatus* Okinawa | ***  0.001 | **  0.004 | **0.138** | ***  0.001 | ***  0.001 | **  0.007 |  |
| S. trispilos Ningaloo | ***  0.001 | **  0.006 | ***  0.001 | ***  0.001 | **  0.003 | **0.077** | **  0.009 |
